# Development and feasibility of a wearable infant wrist band for the objective measurement of physical activity using accelerometery

**DOI:** 10.1186/s40814-018-0256-x

**Published:** 2018-03-01

**Authors:** Alessandra Prioreschi, Thomas Nappey, Kate Westgate, Patrick Olivier, Soren Brage, Lisa Kim Micklesfield

**Affiliations:** 1 MRC/WITS Developmental Pathways for Health Research Unit, Department of Paediatrics, School of Clinical Medicine, Faculty of Health Sciences, University of Witwatersrand, Johannesburg, South Africa; 2 Open Lab, School of Computing Science, Newcastle University, Newcastle upon Tyne, UK; 3 MRC Epidemiology Unit, University of Cambridge, Cambridge, UK

**Keywords:** Infant, Toddler, Physical activity, Measurement, Wearable, Feasibility, Product development

## Abstract

**Background:**

It is important to be able to reliably and feasibly measure infant and toddler physical activity in order to determine adherence to current physical activity guidelines and effects on early life development, growth and health. This study aimed to describe the development of an infant wearable wrist-worn band for the measurement of physical activity; to determine the feasibility of the device data for observational measurement of physical activity and to determine the caregiver reported acceptability of the infant wearable wrist band.

**Methods:**

After various iterations of prototypes and piloting thereof, a final wearable band was designed to fit an Axivity AX3 monitor. Mother and infant/toddler (aged 3–24 months) pairs (*n* = 152) were recruited, and mothers were asked for their child to wear the band with enclosed monitor at all times for 1 week (minimum 3 days). Feasibility was assessed by determining technical reliability of the data, as well as wear time and compliance according to requirements for observational measurement. Acceptability was assessed via questionnaire.

**Results:**

Technical reliability of the Axivity AX3 monitors in this age group was good. After excluding days that did not have at least 15 h of wear time, only 2% of participants had less than three valid days of data remaining, and 4% of participants had no data (due to device loss or data loss). Therefore, 94% of participants were compliant, having three or more days of wear with at least 15 h of wear per day, thus providing enough valid data for observational measurement. The majority (60%) of mothers reported being “very happy” with the safety of the device, while only 8% were “a little worried”. A large majority (86%) of mothers stated that the band attracted attention from others, although this was mostly attributed to curiosity about the function of the band. Most (80%) of participants rated the comfort of the band as “comfortable”, and 10% rated it as “very comfortable”.

**Conclusions:**

The infant wearable band proved to be feasible and acceptable according to the criteria tested, and compliance wearing the band was good. We have therefore provided a replicable, comfortable and acceptable wearable band for the measurement of infant and toddler physical activity.

**Electronic supplementary material:**

The online version of this article (10.1186/s40814-018-0256-x) contains supplementary material, which is available to authorized users.

## Background

Physical activity is known to have beneficial effects on health through the life course [1]. There is a multitude of data showing levels and patterns of physical activity, and the consequent effects on various health outcomes during childhood [2], adolescence and adulthood [3], as well as in the elderly [1, 4]. However, physical activity in infants and toddlers has not been well described [5], and there is little data examining whether physical activity levels or sedentary behaviour during this time have any effect on development, body composition, or health outcomes [2, 6, 7].

Literature in the field of the first 1000 days of life is ever growing, and evidence has mounted showing that many later life diseases and health complications have their origins in the early life periods (preconception, pregnancy and the first 2 years of life) [8]. However, a recent review of the literature on physical activity in the first 2 years of life showed that, of the available studies that had measured physical activity in children under 2 years of age, only six studies described levels and patterns of activity as an outcome and the conclusions were conflicted as to whether infants and toddlers were meeting current physical activity and recommendations or not [5]. It is clear that more observational studies need to be conducted with the specific aim of measuring and describing physical activity levels and patterns, and associations with health outcomes, within this age group. The review by Prioreschi et al. also shed light on the fact that techniques for measuring physical activity in this age group remain inconsistent, and preferred methodologies do not yet exist [5].

Most studies conducted since 2009 measuring physical activity in the first 2 years of life have used some form of accelerometer (digital device for measuring acceleration) [5]. In infant populations, devices used to measure physical activity need to be very small in order to fit the attachment site, yet should still be securely attached and completely safe and free of any choking hazards. Attachment mechanisms should be hypoallergenic and breathable, as well as acceptable to mothers and caregivers alike; and qualitative work has raised concerns around perceived safety, comfort and acceptability of commonly used accelerometer attachments in toddlers [11]. Placement site of these devices is also a consideration in terms of acceptability, as well as data reliability and comparability [5].

Some studies have attempted to address this problem by designing their own bands for securing accelerometers for infant use [10, 12–14]. These four studies have mentioned constructing cloth wrist or ankle bands to secure accelerometers to infants for short measurement intervals; yet, none have described the design of the attachments or the feasibility for future use [10, 12–14]. Furthermore, accelerometers were reported to have fallen out of the cloth band constructed by Mack and Kleinhenz in 1974 in 40% of cases [13]. These attachment mechanisms are thus not transferable to other devices or populations, nor replicable for future use; and the reliability of the data may be questionable if devices are not securely attached or worn for an appropriate amount of time. The few recent studies that have used accelerometery in infants and toddlers have simply used standard attachment mechanisms (such as the waist-worn ActiGraph belt) [5]. However, a study done in Canada using ActiGraphs in toddlers of a similar age (12–35 months) reported that 12% of mothers who were invited to participate declined as they believed that their toddler would not wear the belt [15]. Furthermore, an additional 35% of invited mothers declined due to other reasons, such as being too tired or not being interested. This high proportion of refusals to participate may indicate poor acceptability of the measurement method. Similarly, very low acceptance rates (only 20%) were found in a study measuring physical activity using ActiGraph in toddlers (12–24 months) in Belgium [9].

Poor acceptability in conjunction with data that may not be feasible for use are issues that could deter researchers from conducting physical activity assessments in this population, or may result in poor data quality that is not comparable to other studies. Therefore, the aims of this study are to (1) report on the development of an infant wearable wrist band for the measurement of physical activity, (2) determine the feasibility of the infant wearable wrist band for the observational measurement of physical activity and (3) determine parent/caregiver reported acceptability of the infant wearable wrist band for objective assessment of physical activity.

The following criteria will be used to determine feasibility and acceptability: (1) the majority of participants (> 80%) should wear the band for at least three, but up to seven, days consecutively; (2) the majority of data produced (> 80%) should be technically reliable; (3) the band should fit an Axivity AX3 accelerometer; (4) the band should be worn for the required number of days by the majority (> 80%) of infants and toddlers between the ages of 3–24 months; and (5) the majority of mothers (> 80%) should rate the acceptability of the device as high according to the design specifications—comfort (very comfortable or comfortable), safety (very happy or happy), the buttoning mechanism (easy to unbutton), and drying capability (very quickly or quickly).

## Methods

### Development of the infant wearable band

The design brief was to develop a reusable wrist-worn band for the Axivity AX3 device (logging accelerometer) that was suitable for continuous wear by infant/toddlers aged 3–24 months. Wrist-worn placement was chosen as the most appropriate for infants and toddlers of various ages and developmental stages alike, based on review of the literature [5]. Initial inspiration for the material qualities was drawn from the design of hospital identity tags, for which there has been a shift from traditional hard-edged non-breathable PVC laminate, to fabric-foam composites. Other key considerations included (1) the encapsulation of the Axivity AX3 devices that could be readily inserted and extracted by researchers, but that would not constitute a choking hazard; (2) the mechanism for secure attachment of the band, allowing it to be attached and removed by both researcher and mother; (3) selection of a stitch pattern that complied with standards for minimising risk of injury through ischaemic injury, puncture, choking, swallow, suffocation, strangulation, and overheating (see BS7907:7007 in Code of Practice for the Design and Manufacture of Children’s Clothing to Promote Mechanical Safety); (4) other general product and specific product safety regulations, including nightwear safety regulations for children (see SI 2005 No 1803 and BS EN 14878:2007 in Code of Practice for the Design and Manufacture of Children’s Clothing to Promote Mechanical Safety); and (5) the quick drying capabilities of the material.

**Fig. 1:**
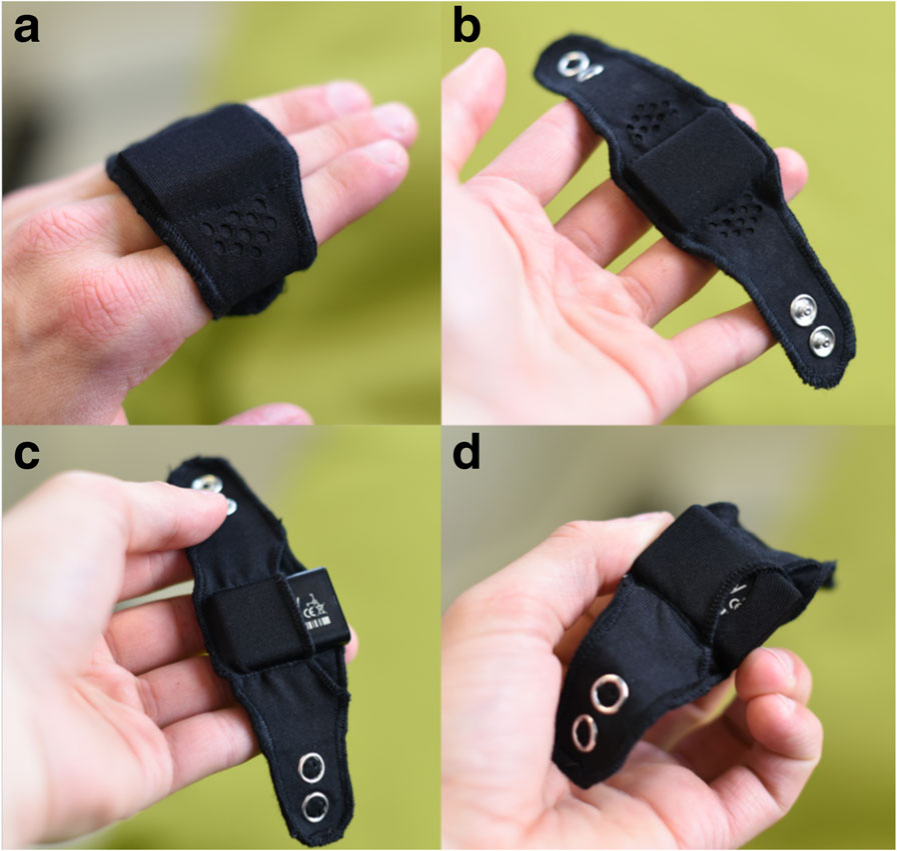
Front (**a**) and back (**b**) of final infant wearable band; and Axivity AX3 insertion (**c** and **d**)

Feedback from parents was solicited for prototype designs when piloted on a small number of infants in Cambridge, UK (*n* = 3) and Soweto, South Africa (*n* = 6). Qualitative feedback from parents related primarily to the security of the bands (parents commented on a range of press stud configuration for fixing bands), and inflexibility of a cotton material used in early designs. The final design (Fig. 1) uses a black EuroJersey polyester-elastane blend material, with a trifold design (three layers of material) for breathability, and to allow for the creation of a “pocket” on the skin-side of the band into which the Axivity AX3 is inserted. To ensure an infant is unable to remove the band themselves, two stainless steel press studs (9.5 mm baby-safe) are oriented in line with the main axis of the band. The final design uses a woolly nylon thread (sealed) that can stretch with the fabric band in a 3-Thread Narrow Overlock pattern. The final designs (full material and component specifications and design patterns) are published under Creative Commons 3.0 BY Attribution licence (see https://github.com/digitalinteraction/openmovement/wiki).

### Feasibility Assessment

#### Participants

Mother and infant pairs (*n* = 152) were recruited at different time points (3 months, *n* = 33; 6 months, *n* = 28; 12 months, *n* = 30; 18 months, *n* = 18; 24 months, *n* = 28) during the first 2 years of life. Mothers and infants were drawn from one of a number of ongoing infant studies at the MRC/Wits Developmental Pathways for Health Research Unit, recruited from the Chris Hani Baragwanath Academic Hospital in Soweto, South Africa. Once included in one of the ongoing studies, mothers were invited to participate in this feasibility study, together with their infants. If the infant had any major diagnosed developmental or physical abnormalities that would preclude them from wearing the device or partaking in normal routine physical activities, they were excluded from the study. All mothers signed informed consent and were free to leave the study at any time. Ethical approval was obtained from the Human Research Ethics Committee of the University of the Witwatersrand (M150632).

#### Study design

Upon maternal consent, the infant was fitted with an AX3 Axivity monitor, (Axivity Ltd., Newcastle-upon-Tyne, UK) worn within the infant wearable band on their left wrist. The monitor is a small raw triaxial accelerometer, weighing 11 g and is waterproof so that it can be worn continuously. Monitors were initialised to collect data at 100 Hz with a dynamic range of + − 8 g. Mothers were asked that their infant wear the band and enclosed monitor at all times for 1 week, and to not remove the band if possible. Mothers completed a logbook recording whether the band was removed at any point and for what reason. After the week period, mothers returned the bands to the research unit and the data were downloaded from the monitors.

#### Wear time, compliance and technical reliability of device data

In order to test the feasibility of the infant wrist band for the observational measurement of infant physical activity, device data were downloaded from the monitors using open source OmGui software (Open Lab, Newcastle University, UK). The number of monitors correctly initialised and downloaded with complete data was recorded and summarised as an assessment of technical reliability. The raw triaxial acceleration data was auto-calibrated to gravity using methods described elsewhere [16]. Vector magnitude was calculated and a high (0.2 Hz) and low (20 Hz) pass frequency filter was applied to the data in order to remove gravity, as well as high frequency noise [17]. Non-wear was identified based on the standard deviation of each axis being below 13 mg for > 1 h [18], and data was summarised at the daily level. Percentage daily wear time was calculated as the number of minutes of wear time per day, averaged across the measurement period. At least 3 days of at least 15 h of wear per day has been reported to be acceptable for analysis of accelerometery data in infants [19], and the number of valid days of data according to this criterion was reported. Feasibility of the device was thus assessed by calculating how many participants were compliant in providing enough valid wear time data to be useable in observation measurement of physical activity.

#### Acceptability of the measurement tool

Acceptability of the infant wearable band according to the infant’s mother was assessed via questionnaire either immediately upon return of the band or shortly thereafter telephonically. The complete list of questions and potential responses are provided as Additional file 1. Mothers were asked to report on various aspects of the infant band namely: comfort, safety, colour, closing mechanism (buttons), fabric, drying speed and perceptions. Comfort of the band was rated in response to the question “How comfortable do you think the band was for your baby?” on a Likert scale ranging from 1 (very uncomfortable) to 5 (very comfortable). The safety of the band was rated on a scale of 1 (I was very happy) to 5 (I was very worried and would not use the band again) in response to the question “Were you happy with the safety of the device and the band?”. Mothers were asked to report their preferred and non-preferred colours from any of the following options: black, blue, red, green, white, pink, other and I do not mind. They were also able to state their preferred or non-preferred colours freely, and more than one colour could be chosen. Mothers were then asked to answer the question “How did you find using the closing buttons on the band?” by selecting one or more of a number of responses provided or by freely describing their response. Mothers were asked to describe the fabric of the band by answering the question “How did you find the fabric material of the band?” by selecting one or more of a number of responses provided or by responding freely. The drying capabilities of the band were assessed by answering the question “How quickly did the band dry if it got wet?” on a scale of 1 (very quickly) to 4 (very slowly). Mothers were then asked: “Did you feel that the band attracted too much attention from other people?” and could respond either “yes” or “no”. Thereafter, they were asked to describe: “How did your baby react to the band?” by selecting one or more of a number of provided responses or by responding freely.

## Results

All data are presented as mean(SD). Of the 152 mother-infant pairs recruited for the study, 146 infant participants provided valid accelerometer data and were included in the feasibility assessment. The mean age of the mothers was 29(6) years, ranging from 19 to 44 years. Just over half (52%) of the infants were males, and there was no difference in distribution of gender by age (*p* = 0.40).

### Wear time, compliance and technical reliability of device data

Technical reliability of the Axivity AX3 monitors was good, with 151 of 152 devices correctly initialised. One monitor was not properly initialised because the battery was not adequately charged before use, and this data was consequently excluded. Four devices were lost by participants and were thus not returned for downloading. All of the monitors that were returned (*n* = 147) were correctly downloaded and provided data. One correctly downloaded file was later lost due to human error. Infants with missing data were significantly older than infants with useable data (*p* = 0.02)—this was due to all the infants who lost their devices being between 18 and 24 months of age. There were no differences according to mother’s age or infant sex.

Since we were unable to assess wear time when data were missing, this assessment was done for those participants with accelerometer data only. Therefore, 146 infant participants were included in the wear time assessment. Average percentage daily wear was 94(20)%. Wear time ranged from 2 to 7 days, with a mean of 6.4(0.7) days. After excluding days which did not have at least 15 h of wear time, three participants (2%) had less than three valid days of data remaining. Therefore, of the participants who had accelerometer data, 98% were compliant, having three or more days of wear with at least 15 h of wear per day, thus providing enough valid data for observational analysis. When considering the whole sample of infants (including those who lost their devices or had missing data), 94% were compliant and provided enough valid data for observational analysis.

### Acceptability

Of the 152 mother-infant pairs recruited, 137 (90%) mothers completed an acceptability questionnaire and were included in the acceptability assessment. Reasons for not completing the questionnaire (*n* = 15) were the following: we were unable to make contact with them (*n* = 11) or because they had lost the bands and were thus unable to answer the questions (*n* = 4). There were no differences in mother’s age, infant age or infant sex between participants included in the acceptability assessment compared to those not included (data not shown). Percentages reported here are thus representative of those who responded and exclude the 10% who were not able to respond.

### Colour

**Fig. 2:**
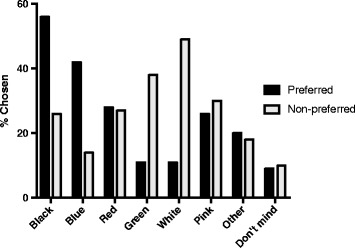
Preferred and non-preferred colours for the infant wearable band

Most participants (98%) preferred a black or blue band, and the non-preferred colours were white (49%) and green (38%) (see Fig. 2). Of those who reported a preferred colour not provided in these options; 13 (8%) would have preferred purple, and 6 (4%) would have preferred yellow, and a small number reported other colours.

### Fabric

Participants were asked to comment on the fabric used to make the band, and various qualities of the fabric, and responses are shown in Table 1. Of those who chose the “other option”, most (70%) stated that they did not notice the speed of drying or tried not to get the band wet. One mother mentioned that she was not happy with the quality of fabric, and another two would have preferred plastic/rubber. One mother mentioned that the band left a mark when it got wet (no further details given). When asked specifically about the drying capabilities of the band, most participants (80%) reported that the band dried very quickly or quickly, while 10% stated that the band dried slowly.

**Table 1:** Mothers’ responses to questions relating to the infant wearable band (each question allowed for multiple responses)

Choose the option(s) that best describes your response to the following questions:	
Fabric	%
Comfortable	96
Quick to dry	70
Started to smell	1
Other	10
Buttoning	%
Easy to close	95
Secure and safe	44
Worried the child would unbutton	35
Do not mind	1
Other	4
Baby’s reaction	%
No difference noticed	66
Curious	61
Ignored the band	15
Tried to take the band off	34
Quickly got used to the band	53
Took a long time to get used to the band	9
No reaction	18
Other	13

### Safety and buttoning

Most (60%) of the mothers reported being “very happy” with the safety of the device, and a further 27% were “happy”, while 8% were “a little worried” and only one mother (1%) was “very worried” (no further information given). Comments regarding the buttoning mechanism of the device are reported in Table 1. Of the 4% who chose “other”, two mothers had not tried to unbutton the band at all, and three had decided to sew the band closed as an extra precaution. One mother was worried about other people unbuttoning the band when she was not around. Of those who were worried that their child might unbutton the band, all but five (14%) had children 12 months or older. There were no additional comments for those who did not perceive the band “safe and secure”, but 40% had also reported they were worried about the baby unbuttoning the band, which may explain the concern.

### Perceptions

A large majority (86%) of mothers stated that the band attracted attention from others. In 74% of cases, people wanted to know what the band was or why it was being worn by the infant. Some people (*n* = 4) presumed the band was a watch; “People asked for the time”. Mostly, mothers reported that “People were just curious”. Mothers were also asked to report how their baby reacted to the band and responses are shown in Table 1. The majority of mothers who listed “other” stated that their baby was trying to chew or bite the band (many were teething). One mother stated that the baby (age 24 months) was very “protective” of the band. One mother stated that her aunt thought that the band was making the baby (aged 3 months) ill, and they therefore removed the band and did not complete the requested 7-day wear period. One mother stated that the band made her baby (aged 18 months) “hyperactive”.

### Comfort

The majority (90%) of participants rated the comfort of the band as “comfortable”, or “very comfortable”. Few (4%) rated the band as “fine”, and the remaining 6% rated the band as “uncomfortable” or “very uncomfortable”. One participant (mother of infant aged 12 months) who rated the band as uncomfortable stated that: “The rubber band (worn by adults) would be better for children”. When asked about the baby’s reaction to the band she stated that: “(my) baby wanted to take the band off, (the) device was itching”, yet the band was not actually removed and this infant achieved 5 days wear time. No other comments were provided for participants who were less satisfied with the comfort of the band.

## Discussion

The aims of this study were to report on the development of an infant wearable band and to examine the feasibility (technical reliability and compliance of the data obtained) and acceptability of this band for the observational measurement of physical activity using accelerometers in infants. To our knowledge, this is the first study of its kind in infants and provides an invaluable resource for researchers who are planning on measuring physical activity at this critical life stage. We have described the process of development of the band and have published full material and component specifications and design patterns; thus allowing for replicability and for modifications to be made if necessary. Overall, the infant band was found to be feasible and acceptable in the Soweto context, with only a few issues noted for future development. Wear time and compliance was very high, as was technical reliability of the data, with only a few (mainly human) errors noted.

The infant wearable band was considered to be feasible and acceptable in this context based on a priori criteria stated in the introduction: (1) the majority of participants (> 95%) wore the band for at least three, but up to seven, days consecutively; (2) the majority of data produced was technically reliable (99% correctly initialised and 100% correctly downloaded); (3) the band was able to fit an Axivity AX3 accelerometer; (4) the band was worn for the required number of days by the majority of infants and toddlers of various ages (91% of 3-month infants, 100% of 6-month infants, 100% of 12-month toddlers, 90% of 18-month toddlers and 88% of 24-month toddlers); and (5) the majority of mothers (80–95%) rated the acceptability of the device as high according to the design specifications—comfort (90%), safety (87%), buttoning mechanism (95%) and drying capability (80%).

In the current study, all but 9 participants (95%) wore the band for three or more days for 15 or more hours per day, with the majority (90%) actually wearing the band for 5–7 days with 99% wear time per day. This is significantly better than the 63% of infants in the Van Cauwenberghe et al. study who provided at least 3 days of data with valid wear time according to less stringent criteria (32% wear time required per day excluding naps compared to 63% wear time required per day in the current study) using waist-worn ActiGraphs [9]. It is noteworthy that of the nine participants who did not provide enough valid data, most (*n* = 6) were due to missing data or devices—indicating that the majority of non-compliance was actually due to loss of devices. It is also important to note that all of the lost devices were from toddlers aged 18 to 24 months, which implies that younger infants with less autonomy were almost 100% compliant. Older toddlers who were able to walk, and were possibly interacting more frequently with other children and with diverse environments, were thus more likely to lose their devices. This suggests that strategies should be developed for better compliance in older age groups. Although some mothers of older toddlers (12–24 months) were concerned about the safety of the buttoning mechanism, the majority of the mothers were happy with the safety of the device itself, and none reported their child being able to take the band off or unbutton the band (although many toddlers were reported to have tried; and it is possible that toddlers who lost their devices had taken them off themselves). This is an improvement over the cloth band constructed by Mack and Kleinhenz in 1974 who reported that out of the five infants tested two devices fell off and had to be replaced [13].

The wearable bands in the current study were generally found to be acceptable in terms of colour, fabric, perceptions, comfort and perceived safety. Some concerns arose around the quality of the fabric, which could potentially give rise to decreased perceived safety of the band if mothers were worried that poor quality may result in the monitor slipping out and becoming a choking hazard. As a result, we have improved the stitching through the use of a professional garment manufacturer. Furthermore, we have concluded that the infant bands would only be used once (one per participant) and disposed of after use, rather than reusing bands between participants (which was done in this feasibility study due to the limited number of bands that could be produced). Two responses in the present study were worth noting. One mother stated that the band made her baby “itchy”, and that the baby wanted to take it off. Since the child (12 months) was unlikely to have voiced this exact reaction, the word “itchy” could be a misrepresentation of the child playing with the band for another reason. However, it is possible that the child was experiencing a dermatological reaction to the band, and in future, mothers should be warned to remove the band if any reaction occurs. A second concerning response was that a family member believed that the band made the child sick, and the band was subsequently removed. Since it is unlikely that a fabric band could cause such an effect, this response is not concerning from a safety point of view, but rather from an acceptability point of view. The Soweto community is made up of a diverse spread of cultures, and many traditional beliefs and norms exist. Furthermore, parenting is often governed by older family members who reside within the home, and traditional views are often enforced [21–23]. This particular mother stated that she was not worried about the band herself, but took it off her baby to appease her elders. Therefore, if this type of perception does exist in certain cultures, it may be considerable when using the band in a larger cohort within the community. This type of response equated to less than 1% of the cohort studied, and the majority of mothers found the bands acceptable, yet it is still worth considering when monitoring compliance in future studies. In a study by Costa et al., South Asian mothers were concerned about the acceptability of the devices for the fathers of their children [11]. Cultural norms and beliefs are therefore vital to assess and consider in relation to feasibility of such measurement tools in the context of the location being studied.

Limitations of the current study include the inability to assess acceptability of the devices in 10% of the participants. Furthermore, the loss of some devices limits the conclusions that can be drawn around wear time and compliance in these infants. We were also not able to determine how devices were lost due to an inability to follow these mothers up, and it is therefore unclear whether these losses have implications for the feasibility of the wrist band. Lastly, we did not quantify how many mothers refused to participate in this study upon recruitment or assess the reasons for declining, thus potentially confounding the results around the acceptability of the wrist band.

## Conclusions

In conclusion, the infant wearable band was feasible for the 24-h observational measurement of infant physical activity over an extended period (7 days), and was largely accepted by mothers in Soweto. Compliance was very high, and most data was reliable and usable. We were able to ascertain potential concerns with the wrist band, and are able to make modifications to future production accordingly. The infant wrist-worn wearable band housing an appropriate accelerometer is thus recommended for future observational measurement of infant and toddler physical activity, in order to provide comparable, objective data, while maintaining safety and acceptability standards as communicated by mothers.

## Additional file

Additional file 1: Acceptability Questionnaire. (PDF 67 kb)

## References

[1] Haskell WL, Lee IM, Pate RR, Powell KE, Blair SN, Franklin BA (2007). Physical activity and public health: updated recommendation for adults from the American College of Sports Medicine and the American Heart Association. Circulation.

[2] Timmons BW, Leblanc AG, Carson V, Connor Gorber S, Dillman C, Janssen I (2012). Systematic review of physical activity and health in the early years (aged 0-4 years). Appl Physiol Nutr Metab.

[3] Janssen I, Leblanc AG (2010). Systematic review of the health benefits of physical activity and fitness in school-aged children and youth. Int J Behav Nutr Phys Act.

[4] Warburton DE, Nicol CW, Bredin SS (2006). Health benefits of physical activity: the evidence. CMAJ.

[5] Prioreschi A, Micklesfield LK (2016). A scoping review examining physical activity measurement and levels in the first 2 years of life. Child Care Health Dev.

[6] LeBlanc AG, Spence JC, Carson V, Connor Gorber S, Dillman C, Janssen I (2012). Systematic review of sedentary behaviour and health indicators in the early years (aged 0-4 years). Appl Physiol Nutr Metab..

[7] Downing KL, Hnatiuk J, Hesketh KD (2015). Prevalence of sedentary behavior in children under 2years: a systematic review. Prev Med.

[8] Barker D (2004). Developmental origins of health and disease. J Epidemiol Commun Health.

[9] Van Cauwenberghe E, Gubbels J, De Bourdeaudhuij I, Cardon G (2011). Feasibility and validity of accelerometer measurements to assess physical activity in toddlers. Int J Behav Nutr Phys Act.

[10] Trujillo-Priego IA, Lane CJ, Vanderbilt DL, Deng W, Loeb GE, Shida J et al. Development of a wearable sensor algorithm to detect the quantity and kinematic characteristics of infant arm movement bouts produced across a full day in the natural environment. Technologies. 2017; 5(39):doi:10.3390/technologies5030039. 10.3390/technologies5030039 PMC5558826 28824853

[11] Costa S, Barber SE, Griffiths PL, Cameron N, Clemes SA (2013). Qualitative feasibility of using three accelerometers with 2-3-year-old children and both parents. Res Q Exerc Sport.

[12] Gravem D, Singh M, Chen C, Rich J, Vaughan J, Goldberg K, et al. Assessment of infant movement with a compact wireless accelerometer system. J Med Devices. 2012;6(2):021013.

[13] Mack RW, Kleinhenz ME (1974). Growth, caloric intake, and activity levels in early infancy: a preliminary report. Hum Biol.

[14] Smith BA, Trujillo-Priego IA, Lane CJ, Finley JM, Horak FB (2015). Daily quantity of infant leg movement: wearable sensor algorithm and relationship to walking onset. Sensors (Basel).

[15] Carson V, Kuzik N (2017). Demographic correlates of screen time and objectively measured sedentary time and physical activity among toddlers: a cross-sectional study. BMC Public Health.

[16] van Hees VT, Fang Z, Langford J, Assah F, Mohammad A, da Silva IC (2014). Autocalibration of accelerometer data for free-living physical activity assessment using local gravity and temperature: an evaluation on four continents. J Appl Physiol (1985).

[17] van Hees VT, Gorzelniak L, Dean Leon EC, Eder M, Pias M, Taherian S (2013). Separating movement and gravity components in an acceleration signal and implications for the assessment of human daily physical activity. PLoS One.

[18] White T, Westgate K, Wareham NJ, Brage S (2016). Estimation of physical activity energy expenditure during free-living from wrist accelerometry in UK adults. PLoS One.

[19] Pitchford EA, Ketcheson LR, Kwon HJ, Ulrich DA (2017). Minimum accelerometer wear time in infants: a generalizability study. J Phys Act Health.

[20] Statistics SA: Census 2011: Census in brief In., vol. Report no.: 03–01-41. Pretoria: Statistics South Africa; 2012: 105.

[21] Evans J (1994). Child-rearing practices in sub-Saharan Africa: an introduction to the studies. The consultative group on early childhood care and development.

[22] Prochner L, Kabiru M, Garcia M, Pence A, Evans JL (2008). ECD in Africa: a historical perspective. Africa’s future, Africa’s challenge: early childhood development in sub-Saharan Africa.

[23] Oheneba-Sakyi Y, Takyi BK (2006). African families at the turn of the 21st century.

