# Supplementary material for: Development and feasibility of a wearable infant wrist band for the objective measurement of physical activity using accelerometery
Source: Pilot Feasibility Stud. 2018 Mar 1;4:60. doi: 10.1186/s40814-018-0256-x (PMC5831201; doi:10.1186/s40814-018-0256-x)
Supplement: Supplementary file 1 — Acceptability Questionnaire. (PDF 67 kb) [file 40814_2018_256_MOESM1_ESM.pdf]

Appendix 1: Feasibility Questionnaire

1. How **comfortable** do you think the band was for your baby? (Tick the box that applies)

Very comfortable	Comfortable	It was fine/ don't know	A little bit uncomfortable	Very uncomfortable

2. Were you happy with the **safety** of the device and the band? (Tick the box that applies)

I was very happy	I was mostly happy	It was fine/ don't know	I was a little bit worried	I was very worried and would not use it again

3. If you had to choose any **colour** for the band what would your preference be? (Tick all those that apply)

- Black
- Blue
- Red
- Green
- White
- Pink
- Other__________(describe)
- I don't really mind

4. Is there any **colour** you do not like for the band? (Tick all those that apply)

- Black
- Blue
- Red
- Green
- White
- Pink
- Other__________(describe)
- I don't really mind

5. How did you find the closing buttons of the band? (Tick all those that apply)

- It was easy to close
- It was not easy to close
- The band provided secure and safe attachment of the device
- I was worried my baby would be able to unbutton it
- I don't really mind
- Other__________(explain)

6. How did you find the fabric material of the band? (Tick all those that apply)

- It was comfortable
- It was uncomfortable
- The band dried quickly when it had become wet
- The band took long to dry when it had become wet
- The band started smelling after a few days
- Other__________(explain)

7. How quickly did the band dry if it got wet? (Tick the box that applies)

Very quickly	Quickly	Neither quick nor slowly	Slowly	Very slowly

8. Did you feel that the band attracted too much attention from other people? (If yes, please comment)

- Yes ______________________(Comment)
- No

9. How did your baby react to the band? (Tick all those that apply)

- My baby did not behave any differently
- My baby was curious about the device
- My baby ignored the device
- My baby wanted to take the device off
- My baby got used to the device quickly
- My baby took a long time to get used to the device
- No reaction
- Other___________(explain)
